# Medical Investigation and Its Practical Results

**Published:** 1888-01

**Authors:** 


### EDITORIAL.

### MEDICAL INVESTIGATION AND ITS PRACTICAL RESULTS.

Among the many important papers presented at the *Ninth International Medical Congress*, held in Washington, that of Professor N. Senn, of Milwaukee, on *Experimental Intestinal Surgery, with reference to the treatment of intestinal obstruction*, probably excited as much interest, and elicted as much favorable comment, as any paper read during the congress. By the results of the work done, in his accustomed pains-taking manner of investigating, Professor Senn, has not only contributed to the advancement of scientific surgery, but has again given evidence of the inaccuracy of the charge that has been made, that Americans make but little original investigation, and are content with utilizing or modifying the results of the labor of other investigators. It is not unnatural that, in in the past, more such study and investigating should have been done in the older countries of Europe, where governments have furnished laboratories and encouraged and aided the investigators, than in America, where, at least until very recently, such work had to be done mainly by individual energy and enterprise; by men who were at the same time engaged in active practice and personally earning the means with which to pursue such investigation. It is much to be desired that the good example set by wealthy citizens of some of the older cities of our country may be followed by others by endowing laboratories and affording greater facilities in connection with medical educational institutions, or otherwise, for the pursuit of scientific research. Among those who are most prone to rail at the alleged unreliability in the practice of medicine are many who could do much by contributions from their abundance to aid in putting the practice of medicine upon a higher plane, if they were as willing to contribute to the means leading to prevention of disease as they are to the support of charitable, or so-called charitable, institutions provided for the care of those who are sick, who are paying the penalty of the violation of the laws of nature—laws violated either by themselves or their progenitors, whether through ignorance of them or through willful disregard of them. Whilst there should be no discouragement offered those who support deserving charitable institutions, maintained for the care of the sick who need and deserve such practical charity, yet it cannot be too often, or too forcibly, impressed upon those thus charitably disposed that there is yet a wider field of usefulness open to them, for all must admit that one serves his fellow man better if he can aid him in avoiding that disease which comes directly or indirectly from violating nature's laws. This can best be done by affording medical men the greatest possible facilities for acquiring full knowledge regarding man in health; the deviations from health, which we are accustomed to designate as disease; the causes and the modes by which disease is produced, and the laws by which nature is governed in her efforts to effect recovery therefrom, that intelligent effort may be made by man to assist nature in her effort. With these data firmly established, the medical man may be regarded as being, first, in the best position to advise his fellow man what to do and what not to do to maintain health, and thus to avoid disease, and in the second place, to aid his recovery from disease which resulted from ignorance or neglect of such knowledge.

In this respect, a wide field is open to the benevolent. So far, in this country, but few of the wealthy, outside of the medical profession, have entered it, but it is honorable, philanthropic, useful. It affords great scope for the most practical form of intelligent charity, and the fact should be better and more widely made known to those who have been favored by fortune, and who wish during lifetime to do good for the benefit of the less fortunate of their fellow men, and to see for themselves the carrying out of their plans, and the good results therefrom, instead of leaving bequests which may or may not accomplish the end sought in leaving those bequests. The field is open to the world, but we hope that there are some among those in Chicago whom Fortune has blessed in a marked degree, and who in other respects have shown in various practical ways their pride in their city and its development, who will be as liberal to this city, which has become so great a medical center, as have been some of the residents of the older cities of our country, and the *Journal and Examiner* will be prompt to give the credit due the one who shall be the first in our city to follow the honorable example so generously initiated, recently, in one of our great seaboard cities.

